# Modern Parisian Practice—The French Therapeutics of the Period

**Published:** 1899-07

**Authors:** Thomas C. Minor


### Modern Parisian Practice.—The French Therapeutics of the Period.

TRANSLATED AND CONDENSED BY THOMAS C. MINOR, M.D.

From the work of Debove and Gouvin, Paris, 1898.

### DISEASES OF THE MOUTH AND PHARYNX.

#### ACUTE STOMATITIS.

Make use of antisepsis for the buccal cavity by means of washes—solutions of boric acid (30:1000), phenic acid (5:1000). Touch the ulcerations daily with tincture of iodine or chromic acid. Give tonic medication.

#### MERCURIAL STOMATITIS.

*Preventive Treatment.*—Care for carious teeth and use antiseptic dentifrice powders. Frequent washing out of mouth with chlorate of potash (5 per 100), or phenic acid solution (5 per 1,000), or boric acid (30 per 1,000).

*Curative Treatment.*—When stomatitis appears suppress mercurial treatment; use antiseptic mouth washes, or rubbing over with boric acid solutions; give iodide of potash internally. If the pains are too acute, use solutions of cocaine, and give the patient some opiate preparations. Give tonic and reconstituent medicine.

APHTHÆ.

Suspend the use of fresh milk, butter, cheese, and submit suspected milk to boiling. Gargle and wash mouth and throat with solution of salicylate of soda (2 per 100). For difficulty in swallowing and eating, paint with solution of cocaine.

ULCERO-MEMBRANOUS STOMATITIS.

Sterilization of drinking water; antiseptic washes for mouth. Chlorate of potash in potion (two to six grains every three hours); also use as gargle. Touch ulcerations with tincture of iodine. Give tonics and reconstituents.

THRUSH.

*Prophylaxis.*—Care should be taken to keep the nursing bottle and nurse's breast clean, as well as the mouth of the child.

*Treatment.*—The basis of treatment should be the alkaloids. Wash out the infant's mouth with Vichy or some similar mineral water. Remove the spots of thrush with a rough handkerchief, previously saturated in a solution of borate of soda.

NOMA—GANGRENE OF THE MOUTH.

Destroy the gangrenous spot with the thermo-cautery. Keep the treatment up if needed until the edges and surface present a healthy, granulating appearance. Use antiseptic washes for mouth. Give tonics and reconstituent treatment.

GLOSSITIS (BLACK TONGUE).

Avoid scraping of the tongue too much, as well as the use of caustics. Use alkaline mouth washes frequently (bicarbonate of soda), touch tongue at moist points with balsam of Peru or with salicylate of soda.

*Epithelial Desquamative Glossitis.*—Treat the cause when possible (gastro-intestinal syphilis).

*Hygiene of the Mouth.*—Care for the teeth, mouth washes of the emollient kinds (slightly alkaline). Forbid the use of tobacco.

*Treatment of Lesion of the Tongue.*—Touch up tongue with boric acid in glycerine or hyposulphate of soda, or balsam of Peru (5 per 100), or salicylic acid (1 to 100). If the tongue is very sensitive paint over with solution of cocaine.

In cases of *leucokeratoses buccales* suppress all causes of irritation of the buccal mucous membrane (tobacco, alcohol, spiced meats, artificial teeth, decayed teeth, etc.) Buccal antisepsis and gastric irrigation. Treat syphilis, gout or diabetes.

*Local Treatment.*—Mouth washes and tepid spraying, frequently repeated, of boric-acid water (5 to 1,000). Touch points as indicated in the treatment of epithelial desquamative glossitis, or with balsam of Peru or solution of lactic acid, bichromate of potash or salicylic acid.

If the lesion continues to undergo evolution and appears about to terminate in an epithelioma, it is necessary to have surgical intervention.

### TUBERCULOSIS OF THE MOUTH AND PHARYNX.

*General Treatment.*—That of tuberculosis.

*Local Treatment.*—Dress ulcerations with a solution of lactic acid (50 to 100), or of phenol (40 to 100). For pains use solutions of morphine, cocaine, antipyrine, or pheno-glycerine.

### BUCCO-PHARYNGEAL SYPHILIS.

Chancres, secondary accidents, demand the administration of mercury (proto iodide pills, corrosive sublimate, inunction of mercury and iodides, or their usage hypodermically). Among myelitics submitted to mercurial treatment it is necessary to recommend severe buccal hygiene—abstinence from tobacco, alcoholic drinks, spiced foods, keeping teeth clean and in sound condition, mouth washes with slightly antiseptic solutions or solutions of chlorate of potash. Mucous patches should be cauterized with nitrate of silver or acid nitrate of mercury every five or six days. If inflammation and pains are too acute, refrain from cauterization and administer gargles with emollient infusion, or use dressings of cocaine.

In case of deep ulceration covered with strong mucus or ulcerations, antiseptic douching, spraying or mouth washes of iodide of potassium or weak iodine are indicated.

In cases of threatening phagadena, give iodide of potassium in large doses (two drachms in divided doses each day.)

PARALYSIS OF VEIL OF SOFT PALATE.

Faradization, strychnia internally or by subcutaneous injections. Among children, tincture of nux vomica in from one to three-drop doses.

PAROTIDITIS.

Antiseptic washes for mouth with solutions of boric acid (30 to 1000), or of chloral (1 to 1,000), antiseptic dentifrices. Simple belladonna ointments or mercurial ointments applied externally over painful regions of neck. Where there is suppuration incise the abscesses and evacuate purulent contents. Internally, give tonics.

